# Ultrasound Measurement of Plantar Fascia Thickness: A Learning Curve Analysis Between a Novice and an Expert Examiner

**DOI:** 10.3390/life16061006

**Published:** 2026-06-15

**Authors:** María Teresa García-Martínez, Javier Martín Llorens, Mª Carmen Ledesma-Alcázar, David Hernández-Guillén, José-María Blasco, Carmen García-Gomariz

**Affiliations:** 1 Ankle and Foot Reserach Advances Group—GAITP, Department of Nursing, University of Valencia, Av. Menéndez y Pelayo 19, 46010 Valencia, Spain; maria.t.garcia-martinez@uv.es (M.T.G.-M.); carmen.garcia-gomariz@uv.es (C.G.-G.); 2 Department of Nursing, University of Valencia, Av. Menéndez y Pelayo 19, 46010 Valencia, Spain; javiermartinll244@gmail.com; 3 Department of Anatomy, Cell Biology and Zoology, Plasencia University Center, University of Extremadura, 10600 Plasencia, Spain; 4 Group of Physiotherapy in the Ageing Process: Social and Health Care Strategies, Department of Physiotherapy, University of Valencia, Gascó Oliag 5, 46010 Valencia, Spain; david.hernandez@uv.es (D.H.-G.); jose.maria.blasco@uv.es (J.-M.B.); 5 Department of Physiotherapy, University of Valencia, Gascó Oliag 5, 46010 Valencia, Spain

**Keywords:** ultrasound, plantar fasciopathy, plantar fasciosis, plantar aponeurosis, plantar fascia, ultrasound measurement, fascia thickness

## Abstract

This study aimed to explore interobserver differences, intraobserver consistency, and temporal changes in ultrasound-based plantar fascia thickness measurements between an experienced musculoskeletal sonographer and a student examiner. Additionally, the study described the student’s longitudinal progression during the measurement acquisition period under supervised conditions. Materials and Methods: This cross-sectional, descriptive observational study included 84 adults, of whom 42 presented plantar fasciopathy and 42 had no history of heel pain. All participants underwent ultrasound assessment of plantar fascia thickness performed by two observers—a novice student and an expert examiner—each obtaining two measurements per participant. Statistical analyses focused on detecting differences between observers and temporal changes in measurement performance. Intraclass Correlation Coefficients (ICCs) were calculated to further explore agreement. Results: In the overall analysis, no statistically significant differences were detected at the group level between student and expert measurements. However, when analyses were stratified by time periods, statistically significant differences were observed during the initial phase (November–December 2024; *p* = 0.026). These differences were no longer consistently detected in subsequent periods. Additionally, interobserver agreement appeared to increase over time; however, this observation is purely descriptive and should be interpreted with caution, as no formal comparisons between ICC estimates were performed and confidence intervals overlap. Conclusions: These findings suggest a progressive reduction in measurement differences between observers over time, consistent with a learning-related effect. However, the results should be interpreted as exploratory and do not constitute evidence of formal interobserver reliability. The findings highlight the potential importance of structured ultrasound training and supervised practice in improving agreement between observers in both clinical and educational settings.

## 1. Introduction

Plantar fasciopathy is a musculoskeletal condition that most commonly affects individuals aged 40 to 60. Diagnosis is primarily based on clinical symptoms and physical examination findings, while imaging techniques such as ultrasound are commonly used as complementary tools for tissue assessment and structural characterization [1]. The condition is particularly prevalent among runners, affecting up to 17.4% of this population [2]. Although plantar fasciopathy has traditionally been associated with an inflammatory process, current evidence indicates that it is primarily degenerative in nature [3,4].

Musculoskeletal ultrasound has become a widely used imaging modality in plantar fasciopathy due to its accessibility, low cost, and ability to evaluate plantar fascia morphology in real time [5]. Ultrasound findings, including increased plantar fascia thickness and altered echogenicity, have been associated with plantar fasciopathy. However, ultrasound assessment is considered operator-dependent, and measurement accuracy may be influenced by examiner training and clinical experience [5,6,7]. In musculoskeletal ultrasound, several studies have described the presence of a learning curve, with novice examiners progressively improving both measurement consistency and agreement performance over time [8,9,10]. Previous research has also compared expert and novice examiners, demonstrating differences in measurement variability, particularly during the early stages of training [6]. This operator dependency is particularly relevant when quantitative measurements, such as plantar fascia thickness, are used for clinical and research purposes [6,11].

Previous studies have examined the reliability and reproducibility of musculoskeletal ultrasound measurements, reporting that both intraobserver and interobserver agreement may vary depending on examiner expertise and training level [6,12]. In this context, understanding how measurement performance evolves with experience is essential to ensure the validity and clinical applicability of ultrasound-based assessments.

In recent years, increasing attention has been paid to ultrasound education and learning curves in musculoskeletal imaging. Previous research has highlighted the importance of structured ultrasound education in improving image acquisition, interpretation consistency, and interobserver reliability [9,13]. Learning curve studies suggest that repeated practice and supervised training may be associated with progressive improvements in measurement performance and agreement between examiners.

Differences between novice and expert examiners have also been reported, particularly regarding anatomical landmark identification and measurement reproducibility [6,12]. In addition, structured musculoskeletal ultrasound training programs have demonstrated improvements in reliability and examiner confidence [13].

Despite these advances, there is limited evidence specifically addressing how measurement agreement and variability evolve over time in plantar fascia ultrasound assessment, particularly when comparing novice and expert examiners under supervised conditions.

Therefore, the aim of this study was to explore interobserver differences, intraobserver consistency, and temporal changes in ultrasound measurement performance between a novice and an expert examiner when assessing plantar fascia thickness. Additionally, the study sought to describe the novice examiner’s learning progression over time under supervised conditions [14,15].

## 2. Materials and Methods

### 2.1. Study Design and Setting

This study is a cross-sectional, descriptive, observational investigation designed to explore interobserver differences, intraobserver consistency, and temporal changes in ultrasound-based measurements of plantar fascia thickness between an expert examiner and a student examiner.

The study was conducted at the University Podiatric Clinic (UPC) of the University of Valencia between November 2024 and April 2025. The study protocol was approved by the Scientific and Ethical Committees for Clinical Research of the University of Valencia. All procedures adhered to the ethical standards outlined in the Declaration of Helsinki, as revised in Hong Kong.

### 2.2. Participants

A total of 85 patients were initially selected. Participants were assigned to either the case group or the control group. The case group included patients presenting heel or plantar foot pain, particularly during the first steps in the morning or after prolonged periods of rest, which are characteristic of plantar fasciopathy. Clinical assessment also considered factors such as physical activity level, type of footwear, recent activity changes, and risk factors such as overweight status or prolonged standing. Diagnosis was confirmed through physical examination, including palpation of the medial calcaneal region, the Lunge test, and the Jack test.

Ultrasound assessment was performed as a complementary diagnostic tool to measure plantar fascia thickness. In symptomatic individuals, increased thickness and structural changes consistent with plantar fasciopathy were observed, while asymptomatic individuals presented values within ranges commonly reported in the literature.

The control group consisted of healthy volunteers with no history of heel pain or plantar fascia pathology. Participants were matched to the study group by age, sex, and activity level. All volunteers underwent a clinical examination to confirm the absence of plantar fasciitis. The control group was included to provide descriptive reference measurements and to contribute to the overall assessment of measurement consistency and was not used as an experimental comparator.

### 2.3. Eligibility and Recruitment

All participants were adults (≥18 years old). Exclusion criteria included gait difficulties, neurodegenerative diseases, prior corticosteroid injections or surgery, or inability to visualize the plantar fascia on ultrasound. All participants provided written informed consent. Subjects were recruited from patients attending the University Podiatric Clinic for podiatric consultation or treatment. An initial screening was performed by a research team member to confirm eligibility.

### 2.4. Ultrasound Assessment Procedure

Measurements were performed by a student examiner and an expert examiner with more than 10 years of experience in musculoskeletal ultrasound. The order of measurements between the student and the expert was randomized to minimize potential order bias, although the sequence was predominantly alternated between examiners, with occasional deviations due to logistical constraints. The expert examiner was blinded to the student’s measurements.

Individuals were assigned to group based on clinical presentation. Due to the nature of the condition, blinding participants to symptom status was not possible. However, data processing was conducted by an independent researcher using anonymized codes.

**Figure 1:**
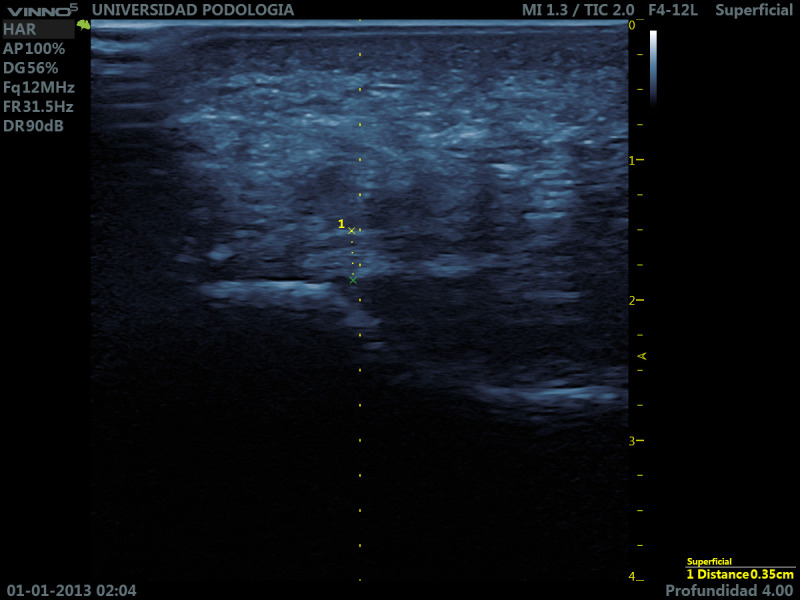

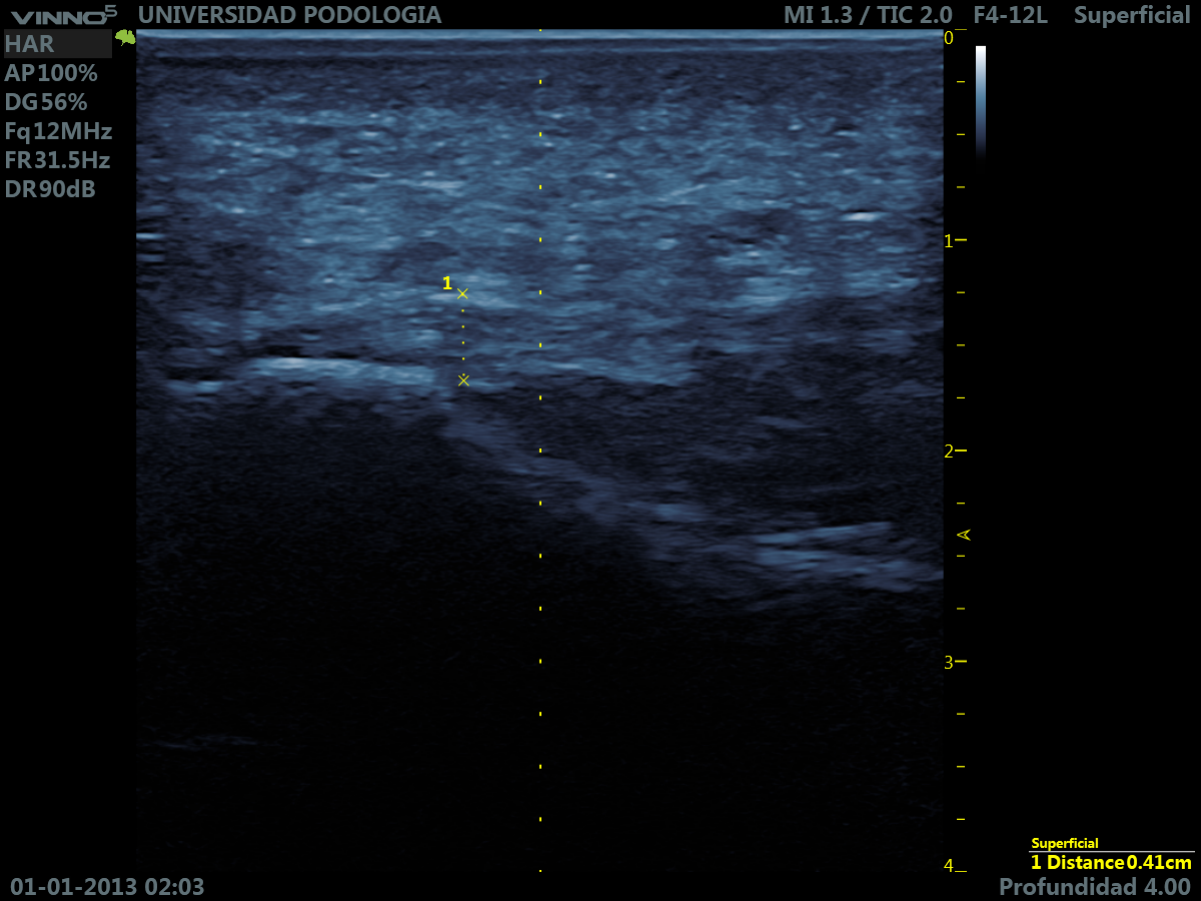
Comparison of the second measurement of the right heel plantar fascia by a student and an expert in a subject with heel pain.

Each participant underwent two consecutive measurements by each examiner under the same conditions. The plantar fascia insertion was identified in longitudinal view, and measurements were performed on frozen ultrasound images (Figure 1). All images were stored for later analysis. The date and order of each measurement were recorded to allow temporal analysis of measurement performance.

Ultrasound acquisition was performed using a Vinno 5 device (VINNO Technology (Suzhou. China) Co., Ltd.) with a high-frequency linear probe (up to 23 MHz). Participants were examined in a relaxed supine position with slight tension applied to the foot to optimize visualization. A standardized musculoskeletal preset was used, and probe positioning and image acquisition were consistent across all participants to reduce variability.

### 2.5. Statistical Analysis

The variables included in the statistical analysis are listed in Table 1. Data analysis was performed using IBM SPSS Statistics version 30.0. Descriptive statistics were calculated for all variables.

Comparisons between student and expert measurements were conducted using paired statistical tests, with a significance level of *p* < 0.05. Reliability was analyzed using the Intraclass Correlation Coefficient (ICC). A two-way mixed-effects model with absolute agreement (ICC (3.1)) was used, as the same examiners performed all measurements. ICC values were interpreted as poor (<0.5), moderate (0.5–0.75), good (0.75–0.9), and excellent (>0.9) [16].

Additionally, temporal analyses were performed to explore changes in measurement differences over time. These analyses were considered exploratory in nature.

No a priori sample size calculation was performed, as the study was designed as an exploratory investigation.

## 3. Results

Of the 85 patients initially recruited, 84 met the inclusion criteria. One patient was excluded because a long-axis ultrasound measurement of the plantar fascia could not be obtained. Statistical analyses were therefore performed on the remaining 84 participants, all adults aged 18–79 years.

### 3.1. Overall Comparison Between Observers

When measurements were analyzed overall, no statistically significant differences were detected at the group level between the student and the expert in either the case or control groups (Table 2).

### 3.2. Agreement Between Observers (ICC)

Interobserver and intraobserver agreement were further assessed using the ICC (Table 3).

Interobserver agreement between the student and the expert was moderate to good (ICC = 0.74), indicating acceptable consistency between observers.

Intraobserver reliability was high in both examiners. Both the expert and the student demonstrated excellent consistency (ICC = 0.94), reflecting a high degree of reproducibility between repeated measurements.

### 3.3. Temporal Variability (Learning Effect)

When analyses were stratified by time periods (Table 4), statistically significant differences between the student and the expert were observed only during the first period (November–December; *p* = 0.026). No significant differences were found in subsequent periods, indicating a progressive reduction in measurement differences over time at the descriptive level.

Similarly, interobserver agreement appeared to increase across time periods; however, this observation should be interpreted as descriptive, as no formal comparisons between ICC estimates were performed and confidence intervals overlap. In the control group, no statistically significant differences were detected across periods, although the first period approached statistical significance (*p* = 0.065), suggesting greater variability during the initial measurement phase.

Interobserver agreement also increased progressively across time periods (Table 5). Lower agreement was observed during the initial phase of the study, whereas higher ICC values were found in subsequent periods. However, no formal comparison between ICC estimates was performed and confidence intervals overlap across time periods. Therefore, this pattern should be interpreted as a descriptive observation rather than a demonstrated improvement in interobserver agreement.

This pattern is consistent with a learning-related effect, whereby repeated exposure and supervised practice may be associated with greater agreement between observers.

The combination of time-stratified analyses and ICC results support a descriptive trend suggestive of a learning-related pattern over time.

### 3.4. Interobserver Variability

Table 6 presents the comparison between the first and second measurements obtained by each examiner. Although both observers showed similar mean values, the expert exhibited lower variability across repeated assessments, indicating greater measurement stability.

### 3.5. Learning Threshold

To explore how measurement differences changed with experience increased, sequential analysis was performed according to measurement order.

In the case group, statistically significant differences between the student and expert were no longer consistently detected after approximately 13 measurements (Table 7).

When considering the overall sample (cases and controls), sequential analysis showed that statistically significant differences were consistently observed up to measurement 34. From measurement 35 onwards, differences were no longer statistically significant (Table 8), indicating convergence between observers.

These findings suggest that approximately 35 measurements may be associated with a reduction in differences between observers when considering the full range of cases. This reflects a progressive reduction in measurement differences over time rather than evidence of formal interobserver reliability. This pattern is consistent with a learning-related effect associated with repeated measurement practice.

These results should be interpreted with caution, as multiple comparisons were performed and the analysis was exploratory in nature.

Statistical significance was defined as *p* < 0.05. Results are presented descriptively due to the exploratory nature of the analysis.

## 4. Discussion

This study evaluated whether there were differences in plantar fascia thickness measurements between a student with no prior ultrasound training and an expert examiner in individuals with plantar fasciopathy.

Although no statistically significant differences were detected at the group level in the overall analysis, a more detailed temporal analysis revealed a different pattern: during the first period of the study (November–December 2024), the measurements did differ significantly between the student and the expert (*p* = 0.026). These differences were no longer consistently detected in the second and third periods. This finding suggests a progressive reduction in measurement differences over time at a descriptive level, consistent with a learning process in which the student gradually improved their ability to identify and measure altered plantar fascia thickness. Although the absence of significant differences suggests comparable measurements, these findings should not be interpreted as evidence of formal interobserver reliability.

In addition to the comparison of mean values, agreement between observers was further explored using the ICC. Interobserver agreement was moderate to good in the overall analysis. In contrast to the initial hypothesis, intraobserver agreement was high in both the student and the expert, reflecting a high degree of reproducibility between repeated measurements in both examiners rather than differences in measurement.

ICC values were calculated using the overall sample to provide a global estimation of agreement between observers. This approach was considered appropriate given that the aim of the study was to explore measurement consistency and its evolution over time, rather than to compare clinical groups. Additionally, the inclusion of the full sample allows a broader range of measurement values, which may improve the robustness and stability of the ICC estimates. While ICC values provide an estimation of agreement, the study was not designed as a formal reliability analysis, and therefore findings should be interpreted with caution. When examined across time periods, ICC values were numerically higher in later stages of the study. However, no formal statistical comparison between ICC estimates was performed, and confidence intervals overlap across periods. Therefore, these findings should be interpreted as descriptive observations rather than as evidence of a demonstrated improvement in interobserver agreement over time. This pattern is consistent with a learning-related effect, whereby repeated exposure and supervised practice may be associated with greater agreement between observers. Nevertheless, these observations should be interpreted with caution as a descriptive finding, given the exploratory nature of the analysis.

Unlike our previous cross-sectional study comparing expert and novice examiners at a single time point, the present investigation focused on the temporal evolution of measurement differences across sequential ultrasound examinations. Importantly, this study was conducted on an independent sample, with no overlap of participants or data from the prior publication. This longitudinal descriptive approach allows the exploration of learning-related patterns over time, which were not addressed in earlier work. Intraobserver variability was also analyzed. Both examiners demonstrated high consistency between repeated measurements, although the expert showed greater precision in identifying anatomical structures, particularly during the initial phase of the study. Although both achieved similar mean values, the higher reproducibility observed in both examiners indicates that variability in measurements was limited and consistent across repeated attempts. This aligns with the existing literature demonstrating a learning curve and the importance of reliability in musculoskeletal ultrasound measurements [9,11]. Previous studies have highlighted the importance of training and supervised practice in development of ultrasound skills among novice examiners [9,10,17]. For example, Vogt and Mayer [9] described progressive improvements among inexperienced operators during early training stages, while Bahner et al. [10] reported that competence can be achieved within a relatively short timeframe with structured teaching. In addition to measurement reproducibility, operator experience may also influence the accurate interpretation of ultrasound images. Previous studies have shown that less experienced examiners are more prone to misidentifying anatomical structures and imaging artifacts, whereas greater expertise improves the ability to distinguish true tissue features from artefactual findings. This aspect is particularly relevant in musculoskeletal ultrasound, where the recognition of phenomena such as anisotropy is essential for accurate assessment [18]. Additionally, differences between novice and expert examiners have been reported in earlier studies, particularly during the initial stages of training [8,15,19]. Larriba-Pérez et al. also evaluated plantar fascia measurements in healthy individuals, although without addressing learning-related effects [8].

The observed effect size (d = 0.51), which falls within the medium-to-large range according to Cohen’s conventions, supports the relevance of the temporal changes observed without implying measurement agreement or reliability. In the absence of comparable studies, these results provide preliminary descriptive evidence of learning-related improvements rather than definitive proof of expert-level accuracy [20,21].

To broaden the descriptive assessment of measurement behavior, we also included participants without plantar fasciopathy. As in the pathological group, no differences were observed among observers when time periods were not considered. However, when analyzing temporal progression, measurements among healthy individuals showed greater variability in the first period (with an effect size of d = 0.96, considered large per Cohen). This can be attributed to the distinct sonographic characteristics of a healthy fascia—striated, fibrillar, homogeneous, with well-defined borders—compared with a pathological fascia, which presents increased thickness, poorly defined borders, loss of fibrillar pattern, and possible Doppler signal. For the student, identifying healthy fascia represented a new perceptual challenge, which explained the initial variability.

Interestingly, measurements in the control group showed greater variability than those in the case group. This may reflect the greater difficulty novices encounter in identifying subtle normal anatomical features compared with the more conspicuous sonographic changes associated with plantar fasciopathy [6,9]. Finally, we explored how the number of measurements influenced the reduction in differences between observers. In the case group, differences were no longer consistently observed after approximately 13 measurements, suggesting an initial improvement in measurement convergence.

When considering the entire sample, this pattern appeared to occur later, with differences no longer consistently detected from approximately 35 measurements onwards. These findings suggest that increasing measurement experience may be associated with a progressive reduction in differences between observers, rather than indicating a precise threshold for competency.

This pattern should be interpreted with caution, as the analysis was exploratory and involved multiple comparisons, and does not provide evidence of a definitive learning threshold or formal interobserver reliability. This study presents several limitations that should be acknowledged. First, although the sample of participants was adequate, the assessment was conducted using only one novice examiner and a single expert examiner. Additionally, although the order of measurements was randomized, in practice, the sequence was predominantly alternated between examiners, with occasional deviations due to logistical constraints. These deviations were limited and are not expected to have substantially influenced the overall results. Moreover, no a priori sample size calculation was performed due to the exploratory nature of the study. Finally, although ICC values were included, the study was not designed as a formal reliability analysis, and findings should be interpreted as descriptive of learning-related changes rather than confirmatory of measurement reliability. Future studies should include a larger number of novice and expert examiners and incorporate dedicated reliability metrics to strengthen external validity.

## 5. Conclusions

Prior ultrasound training may be associated with improvements in measurement consistency and reductions in variability in the assessment of plantar fascia thickness. Greater variability observed in healthy fascia compared with pathological cases may reflect both anatomical differences and the increased difficulty that a novice examiner experiences when identifying subtle normal structures.

This study provides descriptive evidence of a progressive improvement in measurement performance over time, reflecting a learning-related effect in which agreement between a novice and an expert examiner appears to increase with experience. However, these findings should be interpreted as exploratory and do not constitute evidence of formal interobserver reliability. Overall, the results suggest the potential importance of structured training and repeated practice in enhancing ultrasound measurement consistency and agreement in both clinical and educational settings.

## Figures and Tables

**Table 1:** Variable Summary.

Variable	Definition
Group	Case or control based on presence of plantar fasciopathy
ID	Participant identification number
Sex	Male/Female
Height	In centimeters
Weight	In kilograms
Age	In years
Period	Time interval during which measurements were taken
Foot	Foot evaluated
MED.STU1	Student’s first measurement
MED.STU2	Student’s second measurement
Mean. STU	Average of the two student measurements
MED.EXP1	Expert’s first measurement
MED.EXP2	Expert’s second measurement
Mean. EXP	Average of the two expert measurements
Measurement Order	Chronological order of measurements

**Table 2:** Mean plantar fascia thickness measurements obtained by the student and the expert in the case and control groups.

Study Group	*n*	Observer	Mean (mm)	*p*	SD	Effect (d)
Cases	42	Student	4.7	0.419	0.081	0.12
		Expert	4.6			
Control	42	Student	3.3	0.99	0.88	0.0021
		Expert	3.3			

**Table 3:** Interobserver and intraobserver agreement of plantar fascia thickness measurements assessed using ICC.

Comparison	ICC	Interpretation
Interobserver (Student vs. Expert)	0.74 (0.63–0.83)	Moderate–good
Intraobserver Student (Measurement 1 vs. 2)	0.94 (0.91–0.96)	Excellent
Intraobserver Expert (Measurement 1 vs. 2)	0.94 (0.91–0.96)	Excellent

**Table 4:** Comparison of the mean plantar fascia thickness measurements obtained by the student and the expert across time periods in the case and control groups.

Group	Time Period	Observer	*n*	Mean (mm)	*p*	SD	Effect (d)
Cases	Time period 1	Student	22	4.6	0.026	0.06	0.51
		Expert		4.3			
	Time period 2 and 3	Student	20	4.9	0.374	0.08	0.20
		Expert		4.9			
Control	Time period 1	Student	6	4.0	0.065	0.05	0.96
		Expert		3.4			
	Time period 2	Student	23	3.2	0.601	0.11	0.11
		Expert		3.4			
	Time period 3	Student	13	3.1	0.814	0.04	0.06
		Expert		3.1			

**Table 5:** Interobserver agreement across time periods (learning effect).

Period	ICC	Interpretation
November–December	0.67 (0.33–0.84)	Moderate
January–February	0.73 (0.6–0.84)	Moderate–Good
March	0.80 (0.47–0.9)	Good

**Table 6:** Comparison of first and second plantar fascia thickness measurements between expert and student examiner.

Observer	*n*	Measure	Mean (mm)	*p*	SD	Effect (d)
Student	42	M. 1	4.6	0.137	0.038	0.23
		M. 2	4.7			
Expert		M. 1	4.65	0.477	0.051	0.11

**Table 7:** Comparison of plantar fascia thickness obtained by the student and the expert examiner according to measurement in the cases group.

Group	*n*	Observer	Mean (mm)	*p*	SD	Effect (d)
Cases	15	Student	4.56	0.231	0.069	0.32
		Expert	4.33			
Cases	14	Student	4.64	0.165	0.069	0.39
		Expert	4.37			
Cases	13	Student	4.63	0.004	0.043	0.97
		Expert	4.21			

**Table 8:** Sequential *p*-values according to measurement order in the overall sample (cases and controls).

Measurement Order (*n*)	*p*-Value
20	0.001
21	0.031
22	0.048
23	0.025
24	0.021
25	0.027
26	0.036
27	0.023
28	0.017
29	0.018
30	0.012
31	0.014
32	0.019
33	0.014
34	0.011
35	0.087
36	0.130

## Data Availability

The original contributions presented in this study are included in the article. Further inquiries can be directed to the corresponding author. The principal investigator agrees not to use the data for any studies other than those of this project and not to transfer the data to any other potential projects or research teams. We follow the current legislation on the protection of personal data. The results of the study may be published in scientific journals or general publications. However, information concerning participation will be kept confidential.

## Abbreviations

The following abbreviations are used in this manuscript:

UPC	University Podiatric Clinic
BMI	Body Mass Index
ICC	Intraclass Correlation Coefficient
